# Deep Neural Network for Slip Detection on Ice Surface

**DOI:** 10.3390/s20236883

**Published:** 2020-12-02

**Authors:** Kent Wu, Suzy He, Geoff Fernie, Atena Roshan Fekr

**Affiliations:** 1 The Kite Research Institute, Toronto Rehabilitation Institute—University Health Network, University of Toronto, Toronto, ON M5G 2A2, Canada; kentwhf.wu@mail.utoronto.ca (K.W.); shuqi.he@mail.utoronto.ca (S.H.); Geoff.Fernie@uhn.ca (G.F.); 2 Institute of Biomedical Engineering, University of Toronto, Toronto, ON M5S 3G9, Canada

**Keywords:** slip detection, injury prevention, deep neural network, convolution, spatiotemporal feature extraction

## Abstract

Slip-induced falls are among the most common causes of major occupational injuries and economic loss in Canada. Identifying the risk factors associated with slip events is key to developing preventive solutions to reduce falls. One factor is the slip-resistance quality of footwear, which is fundamental to reducing the number of falls. Measuring footwear slip resistance with the recently developed Maximum Achievable Angle (MAA) test requires a trained researcher to identify slip events in a simulated winter environment. The human capacity for information processing is limited and human error is natural, especially in a cold environment. Therefore, to remove conflicts associated with human errors, in this paper a deep three-dimensional convolutional neural network is proposed to detect the slips in real-time. The model has been trained by a new dataset that includes data from 18 different participants with various clothing, footwear, walking directions, inclined angles, and surface types. The model was evaluated on three types of slips: Maxi-slip, midi-slip, and mini-slip. This classification is based on the slip perception and recovery of the participants. The model was evaluated based on both 5-fold and Leave-One-Subject-Out (LOSO) cross validation. The best accuracy of 97% was achieved when identifying the maxi-slips. The minimum accuracy of 77% was achieved when classifying the no-slip and mini-slip trials. The overall slip detection accuracy was 86% with sensitivity and specificity of 81% and 91%, respectively. The overall accuracy dropped by about 2% in LOSO cross validation. The proposed slip detection algorithm is not only beneficial for footwear manufactures to improve their footwear slip resistance quality, but it also has other potential applications, such as improving the slip resistance properties of flooring in healthcare facilities, commercial kitchens, and oil drilling platforms.

## 1. Introduction

Falls are among the leading causes of injuries, especially in the older adult population. Falls result in 646,000 deaths per year globally and pose significant financial burdens on healthcare systems [1,2,3,4]. Falls are induced by loss of balance that is often generated by external perturbations [5]. One of the most prevalent perturbations is slipping, which occurs at the shoe and floor interface when the required walking friction exceeds the existing friction provided by the interface [6,7,8]. Slip-related falls can lead to serious medical complications such as hip fractures and traumatic brain injuries, which contribute to limitations in independent living and lower quality of life [2,9,10]. Slip-resistant footwear can play an important role in preventing slip-induced falls [5,6,7,8]. However, the current standards for measuring the slip resistance of winter footwear are inadequate and have poor biofidelity [2,9,10]. This makes it difficult for footwear manufacturers to optimize their outsole designs and materials properly and to inform clients of footwear performance. Our lab at the KITE Research Institute (Toronto Rehabilitation Institute, University Health Network) introduced a novel human-centered approach called Maximum Achievable Angle (MAA) for testing winter footwear. This test is based on the maximum slope angle that the participant can walk on without slipping [2]. Currently, a trained human observer identifies when slips occur during the test. Although the MAA test shows promising results [11], it is critical to replace human observers with a more reliable and higher-resolution system to assess slips automatically in real time. Based on our current testing experiment, testing five pairs of boots will take about an hour in WinterLab and the observer needs to stay in the cold environment, continuously. Therefore, the rate of errors likely increases over time. In addition, different observers have different learning curves that affect testing results. Using an automated detection system will remove conflicts associated with human errors for slip detection and all footwear will be evaluated with a single consistent algorithm. Since cameras are already in use in the current MAA protocol, it is convenient to use the video data and develop a vision-based solution for this problem. Therefore, the main goal of this paper is to propose a novel deep learning approach to detect slips in real time. Although this new technique can be used in different contexts e.g., monitoring slip accidents in healthcare centers, homes, commercial kitchens, etc., our specific research problem in this paper is to improve the current footwear testing protocol where the detection of slips is an integral part of the algorithm.

## 2. Literature Review

Very few studies were found on slip detection methods, especially on icy surfaces. One reason might be the scarcity of reliable datasets, which is a main obstacle for the development of machine learning models. Okita developed an algorithm for slip detection in robots using Inertial Measurement Units (IMUs). The algorithm was tested on dry and contaminated surfaces [12]. A false negative rate of 20% to 40% and a false detection rate of 5% to 15% were reported in a level walking experiment. Lim et al. presented a slip and trip detection method using a smart phone in the participant’s pocket with data collected in a simulated construction jobsite [13]. Although the device showed 88% accuracy for slip detection, their data were limited to only three workers and 49 simulated slips on a contaminated surface. Trkov et al. [14] proposed an algorithm using five IMUs attached to the lower limb of a single subject. They used a dynamic model for detecting slips in human walking. No numerical accuracy was reported in this paper. Trkov et al. [15] also recently proposed another threshold-based method for slip detection and slipping distance estimation using force sensors and five IMUs. Eight participants were recruited to walk on a wooden platform covered by a soap film to generate slippery conditions. They compared the signals from IMUs with motion capture; however, no detection rate was reported in this paper. Hirvonen et al. [16] proposed a technique to detect sudden movements caused by slips and trips. This method was based on measurements of horizontal acceleration of the trunk. Twenty participants were recruited to walk on an 8 m long contaminated platform to generate 38 slips. They detected 22 out of 38 slips without reporting the false alarm rate. The limitation of this study is that they only detected the slips with a slip distance greater than 5 cm. An insole sensor system was presented by Lincoln et al. [17] to detect the slips using a threshold-based algorithm. For a specific user, the proposed system should be iteratively calibrated to provide a greater than 90% slip detection rate. Recently, Cen [18] proposed a slip detection algorithm with 91% accuracy using nine motion capture reflective markers. Nine healthy adults walked on different slope angles on icy surfaces. Albeit useful, this method can only be used in a lab environment equipped with a 14-camera passive motion tracking system. In addition, the motion capture data are often noisy and incomplete because of calibration error, poor sensor resolution, incorrectly affixed markers, or occlusion due to body parts or clothing. Therefore, much time and effort are required to clean the motion data prior to use.

All these previous studies have some limitations such as the low number of participants, the small size of the dataset and a large number of sensors (e.g., five IMUs), which affect the usability, cost of the system, and possibly high false alarm rates. Most of the studies are based on wearable technology [19,20,21], which might affect the normal gait pattern of the participants in our application. Although there exists no previous vision-based approach to detect slip events, vision-based approaches have been used in human action recognition. For example, Liu et al. [22] proposed a Hierarchical Clustering Multi-Task Learning (HC-MTL) method that alternates between learning and relatedness discovery, iteratively. Their proposed method realized joint action recognition and task grouping. They evaluated their model using realistic datasets such as HMDB51 [23] and UCF101 and showed that the HC-MTL method outperformed other methods for both action grouping and recognition. In addition, Min et al. [24] proposed a Multi-Objective Matrix Normalization (MOMN) method for fine-grained visual recognition. Their proposed system can simultaneously normalize a bilinear representation in square-root, low-rank, and sparsity. They evaluated the system using five benchmark datasets, including the MPII human pose dataset [25], for which the system achieved a Mean Average Precision (mAP) of 34.3%. These studies are just a few examples in human action recognition. However, to date, there exist no vision-based methods for slip detection, specifically. Therefore, in this paper, for the first time, a novel vision-based deep learning method is proposed to detect the slips in real time. The system was trained by data from 18 participants walking uphill and downhill on two types of ice surfaces (dry and wet) with different slope angles. Although the model was mainly developed to improve the MAA method in ranking the winter footwear, it could also be readily applied in other areas where slip incidence is important, such as hospitals, commercial kitchens, and oil drilling platforms.

To summarize, the novelty of this paper is to first provide a new dataset of slip and no-slip events considering different types of footwear, walking directions, slope angles, and different types of ice with real, human participants. In our dataset, the participants were asked to walk normally without any interference on their normal walking pattern. The second contribution is the proposed deep learning model, which to our knowledge has never been used for this specific application.

## 3. Materials and Methods

### 3.1. Testing Methodology

**Figure 1:**
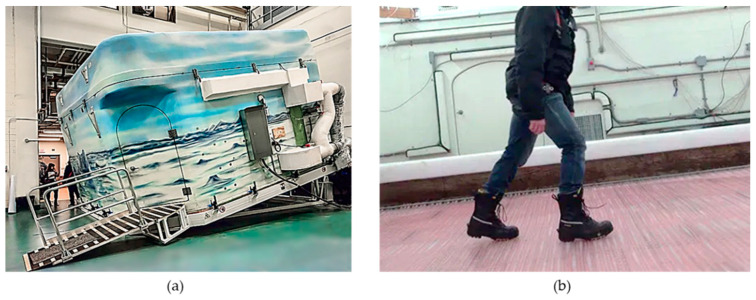
WinterLab at the KITE Research Institute (**a**) outside view and (**b**) inside view (glycol pumped through tubes below the ice surface).

In 2015, researchers at KITE Research Institute, Toronto Rehabilitation Institute (TRI) established a new testing method that measures the maximum achievable incline during walking on icy surfaces. This angle was used to measure the Coefficient of Friction (COF) values for each footwear [2,9]. This human-oriented protocol is run in WinterLab, which has an ice floor, a snow maker, and air and ground temperature controllers. This self-contained winter environment can be tilted to progressively greater inclines from 0° to 15° as participants walk up and down slopes to test footwear. In WinterLab, we can simulate two types of ice surfaces: Wet/melting ice and dry/bare ice. In the wet ice condition, the air temperature is held to between 7 °C and 10 °C while the ice surface temperature is held between −0.1 °C and 0.8 °C. This condition simulates the situation where there is a very thin layer of water on the ice. For the dry ice condition, the air temperature is between 2.5 °C and 3.5 °C with the surface temperature between −4 °C and −3.5 °C, which simulates a colder winter day. The temperature of the ice is controlled using glycol pumped through tubes below the ice surface. Figure 1a,b shows the outside and inside the WinterLab, respectively. Participants are secured with a harness attached to a fall-arrest system connected to a robotic overhead gantry that follows the position of the subjects to maintain itself directly above them. This system minimizes the risk of injury from falling in the event of a slip-and-fall incident. In the current footwear testing protocol, the slope angle of the walkway is progressively increased by 2° until the first failure. An angle is considered as the fail angle if the participant could not initiate gait or if both feet slipp simultaneously while traversing the slope. Thus, an angle is considered as the MAA if the participant can walk successfully on two out of three trials at this angle, and the participant fails on two out of three trials at the (angle + 1)° [26]. In this protocol, a trial is defined as a single walk up or down in WinterLab. Separate outcomes are recorded for the uphill, downhill, dry, and wet ice conditions. The slips are detected by a trained human observer who sits inside the lab.

### 3.2. Data Collection

**Figure 2:**
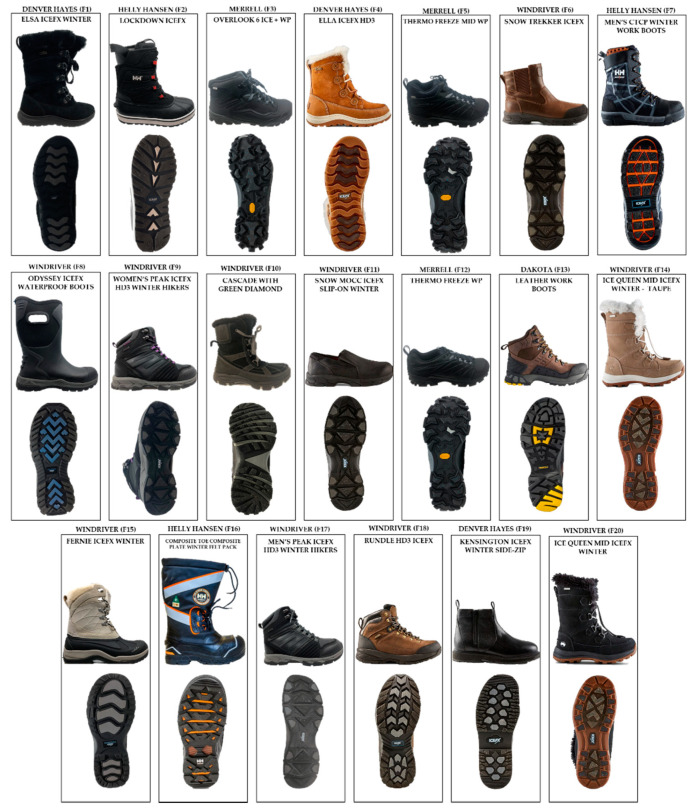
Twenty types of winter footwear considered in this study.

A GoPro Hero 3 camera with 30 fps was used to record a sample of 360 trials with a balanced distribution of the two classes (pass/fail). The data were collected from 18 participants (10 Males and 8 Females) wearing 20 different styles of winter footwear shown in Figure 2. The demographic information of the participants is listed in Table 1. Exclusion criteria included people with musculoskeletal and cardiopulmonary disorders, orthopedic disease, and any other condition that would affect mobility.

Ethics approval for the study was obtained from the KITE-TRI-UHN Research Ethics Board, and participants gave written informed consent prior to study participation. Most of the recorded videos lasted from 100 (~3 s) to 300 (10 s) frames in total. Despite inevitable fish-eye effects by GoPro cameras, a relatively narrow Point of View (POV) had been applied to the camera to offset such perspective distortion. In this paper, our model will classify the types of slip into: Maxi-slips (hazardous), midi-slips, and mini-slips while the other class represents normal walking. This classification is based on the slip perception and recovery of the participants. During mini-slips, the participants did not feel the slip events but the trained human observer could recognize them after checking the videos frame by frame. During midi-slips, the slips events were recovered without major gait disturbances and in maxi-slip events, the slip recovery involved large corrective responses and the participants were close to a fall [27,28].

### 3.3. Neural Network Architecture

**Figure 3:**
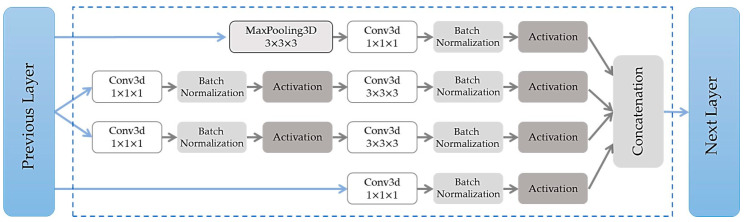
Inception Module of the I3D architecture. Batch normalization and activation layers are in the same shape as their input data.

For our video binary classification problem, we have used a pre-trained model proposed by Carreira et al. in [29]. This model, called “Inflated 3D ConvNets” (I3D), has been used in the action recognition dataset, with 71.1% accuracy in classifying 400 types of human actions with RGB data only (RGB-I3D) [30]. After pre-training on ImageNet and Kinetics, RGB-I3D models provided accuracy of 95.6% and 74.8% on UCF-101 [30] and HMDB-51 [23] datasets, correspondingly. The applied I3D model was based on the Inception-v1 with batch normalization [31] with inflating filters, and pooling kernels into 3D (see Figure 3). The final model consists of a very deep, naturally spatiotemporal classifier [29]. Particularly in Figure 3, features are extracted from video inputs with max pooling operations and convolutional kernels with different sizes, resulting in a simple yet effective parameter selection.

Since this model showed promising results in classifying different types of human actions using video files, in this paper, we have used the RGB-I3D network, which takes advantage of pre-training on both ImageNet and Kinetics. The performance of the pre-trained models by Kinetics was higher than previous 3D ConvNets (C3D), although C3D was trained on a larger number of video files [29], and even when combined with Improved Dense Trajectory (IDT) [29]. This may be due to a better quality of Kinetics and the I3D architecture.

With OpenCV, we uniformly extracted 64 frames out of each sample with 224 × 224 resolution. The preprocessed data were then used to retrain the I3D Neural Network based on the pre-trained weights from ImageNet and Kinetics. In particular, low-level motions at human posture in our dataset are the key characterization of the slip events. This property should be taken into account for feature learning and inference throughout the deep convolutional architecture of I3D.

### 3.4. Experiment Setting and Model Configuration

The performance of the proposed model is evaluated based on five different experiments as follows:

1. 60 trials of maxi-slips and 60 no-slip trials,
2. 60 trials of midi-slips and 60 no-slip trials,
3. 60 maxi-slips, 60 midi-slips and 120 no-slip trials,
4. 60 mini-slips and 60 no-slip trials,
5. 180 slips of all three types and 180 no-slip trials.

**Figure 4:**
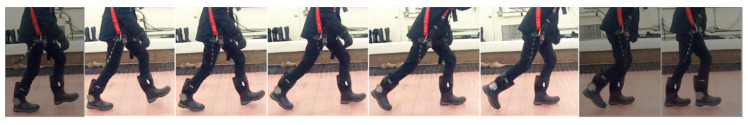
A sample of mini-slip on slope angle 11° and dry ice surface. The slip starts from the second frame and lasts for five frames on the right foot.

A sample of mini-slip is presented in Figure 4. The slip starts from the 2nd frame on the right foot and lasts for five frames on the dry ice surface. This example is from sub2′s trial when wearing F8. Note that the no-slip trials are the same for scenarios 1, 2, and 4. To summarize, we arranged scenarios with individual subsets of slip data, a scenario with full data, and one with fair detection, in experiment 3. We ran the RGB-I3D pre-trained model for binary classification for each scenario. Considering the small-scale training data, we used 5-fold cross validation to evaluate the performance of each model for all scenarios. As our goal is essentially to classify slip and no-slip activities, a binary cross entropy is introduced as the loss function as follows:

(1) $$f\left(x\right)=-\frac{1}{N}\overset{N}{\underset{i=1}{\sum }}y_{i}\log\left({\hat{y}}_{i}\right)+\left(1-y_{i}\right)\log\left(1-{\hat{y}}_{i}\right)$$

where *N* is the training sample size, $y_{i}$ is the ground truth label for the class slip and no-slip, as 0 and 1, respectively, and ${\hat{y}}_{i}$ denotes the corresponding predicted value. We seek to minimize this loss function during the training regime. The smaller the difference between the true label and the probability, the lower the loss function is. In this paper, Adaptive Moment Estimation (Adam) [32] was used as the optimizer, which uses estimations of the first and second moments of gradient to adapt the learning rate for each weight of the neural network. Different publications [33,34] showed the feasibility of Adam to tackle problems on transfer learning. However, in experiment 4, the standard Stochastic Gradient Decent (SGD) [35] yielded a better and more consistent result than Adam.

In experiment 4, we aim to detect the mini-slips that are very close to no-slip trials. When confronted with this challenge, the infrequent large gradients that induce a large update to the weights will be phased out quickly with the exponential moving average algorithm in Adam, leading to poor convergence [36,37]. The learning rate has been set to 0.001 for all experiments.

To prevent overfitting, we used a dropout layer and an early stopping mechanism, which terminates the training process automatically once the loss function stops improving after a pre-determined number of epochs (patience). We empirically set the patience value to be 5 epochs in experiments 1–3. Due to the significant resemblance for the two classes in experiment 4, the patience value was set to 10 in this scenario, and accordingly set to 8 in experiment 5, which comprises the entire dataset.

## 4. Results

### 4.1. Data Analysis

**Figure 5:**
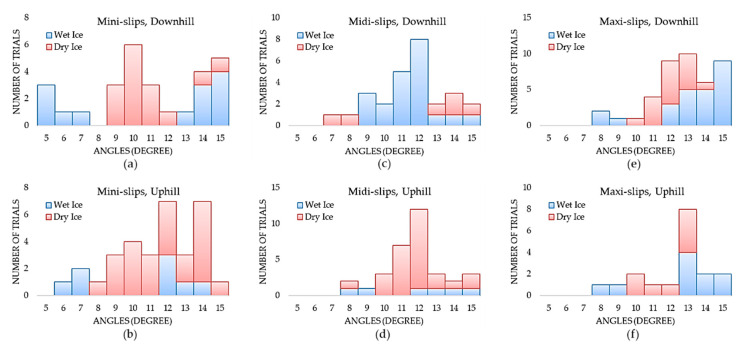
The total number of mini-slips in walking (**a**) downhill and (**b**) uphill, the total number of midi-slips in walking (**c**) downhill and (**d**) uphill, and the total number of maxi-slips in walking (**e**) downhill and (**f**) uphill on dry and wet ice surfaces.

**Figure 6:**
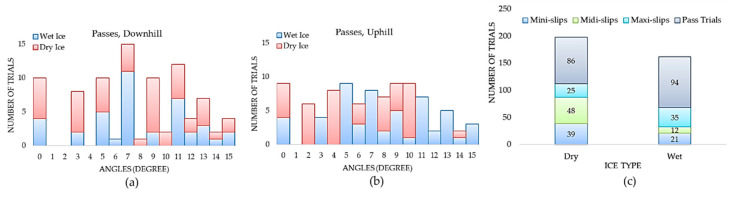
The total number of passed trials in walking (**a**) downhill and (**b**) uphill, on dry and wet ice surfaces, and (**c**) total number of trials on dry and wet ice surfaces.

We developed a very first slip detection dataset of 360 video clips with a balanced distribution of slips (180 trials) and no-slips (180 passed trials). In our testing procedure, 18 participants wore 20 different footwear between sessions. Footwear anti-slip characteristics (Coefficient of Friction (COF)) are mainly ascribed to the outsole technology, design, and material. Figure 5 illustrates the total number of mini, midi, and maxi slips considering different ice conditions, angles, and walking direction. All slips occurred at angles greater than 5° and the maxi-slips started from slope angle 8°. The maxi-slips mostly occurred in walking downhill (Figure 5e). Regardless of walking direction, mini-slips and midi-slips are more frequent on dry ice, whereas the frequency of maxi-slips does not differ significantly between wet and dry ice. Figure 6a,b shows the “No-slip Trials” distribution over different angles for walking downhill and uphill, respectively.

**Figure 7:**
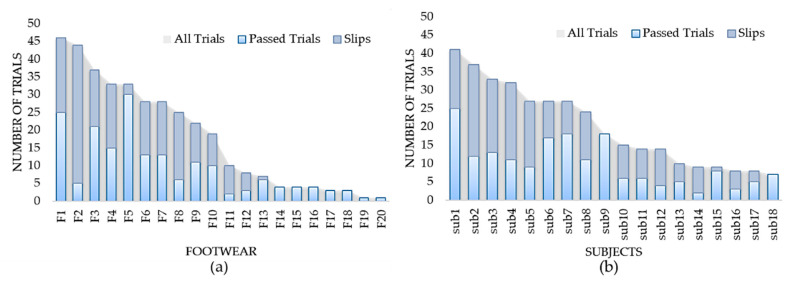
The total number of trials performed by (**a**) each type of footwear and (**b**) each participant.

We had an almost equal number of passes for dry and wet ice conditions with 86 and 94 trials, respectively. Figure 6c also shows that in our dataset, the slip events occurred more on dry ice (112 out of 180 slips) than on wet ice (68 slips out of 180). All footwear and participant contributions to our dataset are shown in Figure 7a,b, correspondingly. The grey shaded regions in these figures indicate the total number of trials for each footwear and participant. For example, F1 had the most contributions to the dataset with 46 trials (21 slips and 25 passes) while F20 and F19 had the least contributions in our dataset with only one no-slip trial.

### 4.2. Model Evaluation

We evaluate the architecture’s performance in the five concise experiments defined in Section 3.4. The classification metrics such as accuracy, recall, specificity, and F1 score are obtained as follows:

(2) $$Recall/Sensitivity=\frac{T_{p}}{T_{p}+F_{n}}\;Specificity=\frac{T_{n}}{T_{n}+F_{p}}$$

(3) $$Accuracy=\frac{T_{p}+T_{n}}{T_{p}+T_{n}+F_{p}+F_{n}}\;F1\;Score=\frac{2T_{p}}{2T_{p}+F_{p}+F_{n}}$$

where $T_{p}$, $T_{n}$, $F_{p}$, and $F_{n}$ denote true positives, true negatives, false positives, and false negatives respectively. We have also computed the AUC with the trapezoidal rule as follows [38]:

(4) $$AUC=\sum_{i}\frac{1}{2}\left(TPR_{i}+TPR_{i-1}\right)\left(FPR_{i}-FPR_{i-1}\right)$$

where *TPR* refers to the true positive rates, and *FPR* refers to the false positive rates.

#### 4.2.1. Record-Wise Cross Validation Analysis (5-Fold)

**Figure 8:**
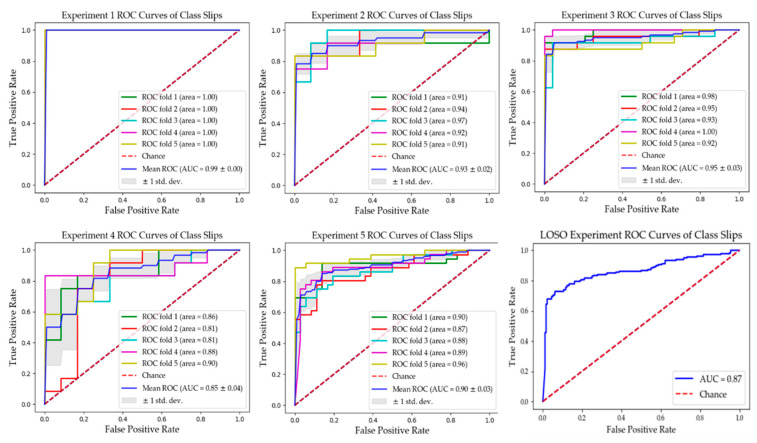
Receiver Operating Characteristic (ROC) curves for our experiments.

**Figure 9:**
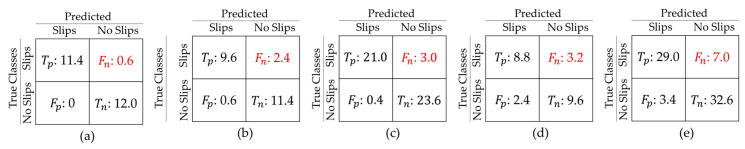
Confusion matrices of average prediction results from 5-fold cross validation for (**a**) Experiment 1, (**b**) Experiment 2, (**c**) Experiment 3, (**d**) Experiment 4, and (**e**) Experiment 5.

Table 2 summarizes the results for all experiments where most reach over 86% accuracy rates. However, a lower accuracy rate of 77% for experiment 4 was obtained, given the difficulty of detecting mini-slips—the trained human observer could sometimes find them uncertain to identify as well. Notably, our model could detect the maxi-slips with high accuracy of 97%. Overall, the model discrimination ability is clearly associated with slip severity as expected. It is worth noting that the specificity rates suppress the recall rates in all experiments, implying the learning of the passed (no-slip) class is slightly better than the slip class. This could be intuitively explained by the monotonic and distinguishable pattern of human walking, in contrast with complicated postures during slip events. Figure 8 shows the Receiver Operating Characteristic (ROC) curves for all experiments. This figure and Table 2 demonstrate very high areas under curve (AUC) values for all experiments. The average true positive ($T_{p}$), true negative ($T_{n}$), false positive ($F_{p}$), and false negative ($F_{n}$) from 5-fold cross-validation are brought in confusion matrices in Figure 9. The False Negative Rates (FNRs), which are critical in our application, are 5%, 20%, 12.5%, 26.7%, and 19.4% for experiments 1 to 5, respectively. Experiment 4 had the highest FNR, where the model distinguishes between mini-slips and no-slips; this result is consistent with our prior hypothesis that it may be challenging to accurately identify mini-slips even using human observation.

#### 4.2.2. Subject-Wise Cross Validation Analysis (Leave One Subject Out)

**Figure 10:**
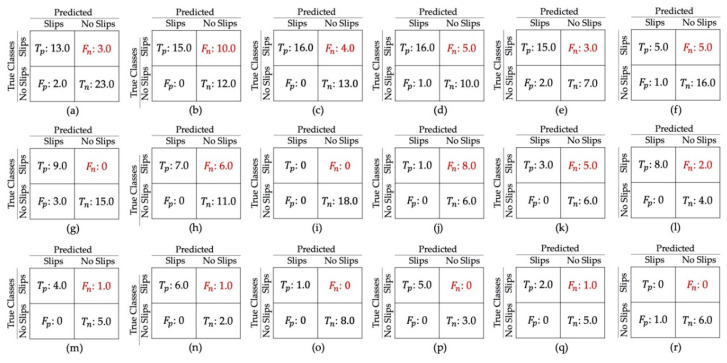
Confusion matrices of Leave-One-Subject-Out (LOSO) results: (**a**–**r**) from sub1 to sub18, respectively.

In our human-centered footwear testing protocol, each footwear is tested by different human participants. Therefore, it is important to evaluate the models using a subject-independent cross validation technique, i.e., Leave-One-Subject-Out (LOSO). In this method, the data of one subject is selected for testing purposes while the other subjects’ data are used for training the model. This procedure is repeated until all the subjects have been used as the test dataset. We want to simulate a scenario when new subjects join our footwear testing program, and to determine if the model is capable of differentiating slip/no-slips from different gait patterns. In our dataset, we had 18 participants, therefore the LOSO validation provides us with 18 different classification parameters. We have used LOSO for experiment 5 and the results are shown in Figure 10 and Table 3. The average accuracy was 84% ± 13% which is 2.3% lower than 5-fold cross validation. This drop in the performance might be due to the fact that in 5-fold cross validation, records from the same subject are present in both training and test folds. In addition, the unbalanced data from our 18 subjects, shown in Figure 7b, may lead to overestimation in 5-fold cross validation.

**Figure 11:**
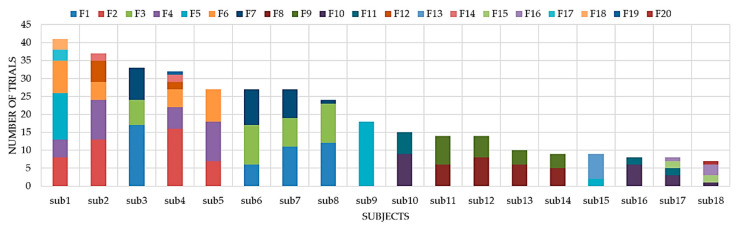
The total number of trials performed by each subject with all 20 boots.

Since, in LOSO cross validation, the test data in each iteration may be unbalanced with class labels, it is necessary to evaluate the specificity, sensitivity, and F1 score in this type of validation. As depicted in Figure 11, every subject used different numbers of footwear to produce slips and no-slips data. For example, sub1 used 6 pairs of boots (F2, F4, F5, F6, F17, and F18) to provide 25 and 16 trials with and without slips, respectively (see Figure 7b). It is worth noting that sub9 and sub18 only contributed to the “no-slip” class, thus $T_{p}$ = $F_{n}$ = 0 for these two participants (Figure 10). For sub9, the model that was trained by the other 17 subjects could successfully distinguish all no-slip data without any false positives (specificity 100%). However, for sub18, one of the no-slip trials is misclassified as “slip”, resulting in specificity of 85% shown in Figure 10r. The misclassification shows the false negative and false positive cost of the proposed model. The high specificity for both subjects indicates a rare occurrence of false positives and successful classification for no-slip trials. The worst-case accuracy, which seems to be an outlier in our data, was achieved for sub10. All “no-slip” trials for this subject are detected correctly (specificity 100%); however, eight out of nine slips are misclassified, which leads to an F1 score of 20%.

**Figure 12:**
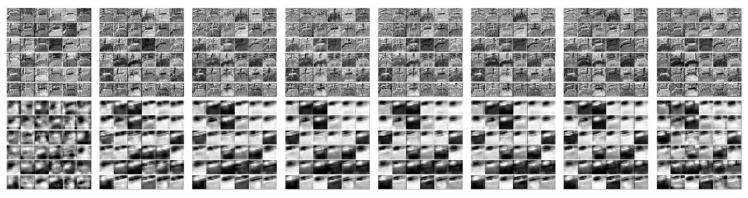
Features maps when feeding a sample video of maxi-slip to the retrained I3D model. The top row shows some features extracted in layer 32, and the bottom row is some features from layer 123.

**Figure 13:**
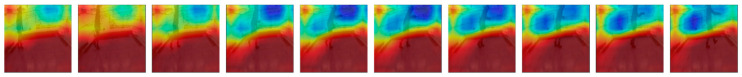
Class Activation Map (CAM) on a sample video of maxi-slip.

Some feature maps obtained from certain layers of our trained RGB-I3D model are shown in Figure 12. Even though the data still outline the slip activity in the bottom layer of the model, it becomes completely unrecognizable by a human once it gets deeper. The “blurry” features may still provide a useful feedback for the model to make its prediction. For simplicity, in Figure 13 we portrayed a Class Activation Map (CAM) before the final SoftMax layer to visualize the attention of our neural network.

One concern is that the model might focus on the feature when participants recover from the maxi-slips by using a rope. However, such rope grabbing action can also happen at the end of no-slip trials where the participants grabs the rope after the last step at the end of the walkway. It was observed that the model can properly localize the event and region of interest both spatially and temporally.

## 5. Future Work

### 5.1. Coverage Limitation

Vision-based detection is often limited by the coverage of the cameras. Although a single static GoPro camera was used in our current dataset, to improve the model we can install cameras at other positions with different frame rates, POV, video resolutions, and even lights. Experiments with fusion of these variables should lead to a robust model that sends warnings in time from different placements and conditions in a room.

### 5.2. Data Processing and Population

Given the small sample size used, data augmentation [39] is also a technique to potentially enhance model generalizability. By imposing additional transformations onto original data, such as cropping, flipping, rotation, etc., more data can be generated to effectively reduce possible overfitting. However, it requires meticulous processing for our dataset, as slip characteristics are informative for learning and we do not want to lose such information. For example, the short contact with the ground surface in mini-slips seems to be the only criterion from a human observer’s standpoint. In addition, since the current participants of this study are young healthy adults, more data will be collected from people with wide range of age to evaluate the model. The current footwear testing is a longitudinal program at KITE Research Institute and this model will be improved over time with data from a larger population and different footwear.

### 5.3. Real Time Detection

Real time slip detection would be very helpful for the efficient operation of WinterLab. Although the current algorithm can only detect the slips in every 64 frames, it is possible to store a cache of previous features and make the classification decision [40] even faster. In the future, we will implement our model in AWS DeepLens, the world’s first deep learning enabled video camera [41] to detect the slips in real time.

## 6. Conclusions

In this paper, we present a deep three-dimensional convolutional neural network architecture capable of detecting slips during human walking in winter conditions using a vision-based approach. We formed an original dataset, consisting of 360 video clips of 18 participants wearing 20 styles of footwear. Retrained by this dataset, our system demonstrated an overall slip classification accuracy of 86%, sensitivity of 81%, and specificity of 91% when using 5-fold cross-validation. In addition, the system demonstrated that the detection accuracy is correlated with the slip severity. The maxi-slips were detected with the highest accuracy of 97%, whereas the mini-slips were detected with the lowest accuracy of 77% among all cases. The average accuracy achieved for LOSO validation was 84%. These results demonstrated the system’s capacity to detect slips of various sizes in realistic and varying environmental conditions. The proposed system can be readily applied to detect slips in real-world winter conditions and other slip-prone scenarios. For example, the system can be implemented in healthcare environments to detect the slips of patients and staff. The prompt and automatic detection provided by the system will allow fast interventions which could reduce the injury rates. The system’s ability to maintain high performance with various footwear styles and flooring conditions contributes to the practicality of its application in preventing injuries, especially for elderly.

## Figures and Tables

**Table 1:** Participants’ demographic information.

Subjects ID	Gender	Age	Height (cm)	Weight (kg)
sub1	M	35	175	75
sub2	M	21	165	54
sub3	M	20	183	75
sub4	M	21	172	70
sub5	M	35	175	75
sub6	M	20	188	82
sub7	M	25	188	93
sub8	M	21	177	71
sub9	M	23	188	106
sub10	F	22	162	54
sub11	F	21	167	55
sub12	F	21	165	54
sub13	F	22	162	54
sub14	F	21	155	62
sub15	M	30	192	56
sub16	F	23	179	84
sub17	F	26	170	62
sub18	F	32	167	66

**Table 2:** Five-fold cross validation results for all five experiments.

Experiments	Accuracy	Sensitivity	Specificity	F1 Score	AUC
Experiment 1	0.97 ± 0.03	0.95 ± 0.07	1.00 ± 0.00	0.97 ± 0.04	0.99 ± 0.00
Experiment 2	0.88 ± 0.05	0.80 ± 0.11	0.95 ± 0.04	0.86 ± 0.06	0.93 ± 0.02
Experiment 3	0.93 ± 0.03	0.88 ± 0.05	0.98 ± 0.02	0.92 ± 0.03	0.95 ± 0.03
Experiment 4	0.77 ± 0.04	0.73 ± 0.10	0.80 ± 0.11	0.76 ± 0.05	0.85 ± 0.04
Experiment 5	0.86 ± 0.04	0.81 ± 0.08	0.91 ± 0.03	0.85 ± 0.04	0.90 ± 0.03

**Table 3:** LOSO results on experiment 5.

Experiments	Accuracy	Recall	Specificity	F1 Score
Sub1	0.88	0.81	0.92	0.84
Sub2	0.73	0.60	1.00	0.75
Sub3	0.88	0.80	1.00	0.89
Sub4	0.81	0.76	0.91	0.84
Sub5	0.81	0.83	0.78	0.86
Sub6	0.78	0.50	0.94	0.63
Sub7	0.89	1.00	0.83	0.86
Sub8	0.75	0.54	1.00	0.70
Sub9	1.00	NA	1.00	NA
Sub10	0.47	0.11	1.00	0.20
Sub11	0.64	0.38	1.00	0.55
Sub12	0.86	0.80	1.00	0.89
Sub13	0.90	0.80	1.00	0.89
Sub14	0.89	0.86	1.00	0.92
Sub15	1.00	1.00	1.00	1.00
Sub16	1.00	1.00	1.00	1.00
Sub17	0.88	0.67	1.00	0.80
Sub18	0.86	NA	0.86	NA
Average	0.84 ± 0.13	0.72 ± 0.23	0.96 ± 0.07	0.79 ± 0.19
